# The impact of multi-morbidity on disability among older adults in South Africa: do hypertension and socio-demographic characteristics matter?

**DOI:** 10.1186/s12939-017-0537-7

**Published:** 2017-04-08

**Authors:** Philippa Waterhouse, Nele van der Wielen, Pamela Chirwa Banda, Andrew Amos Channon

**Affiliations:** 1 Faculty of Wellbeing, Education and Language Studies, Open University, Milton Keynes, UK; 2 Department of Social Statistics and Demography, University of Southampton, Southampton, UK; 3 Department of Demography and Population Studies Programme, University of the Witwatersrand, Johannesburg, South Africa; 4 Ministry of Education, Provincial Education Office, Lusaka, Zambia; 5 Centre for Research on Ageing, University of Southampton, Southampton, UK

**Keywords:** Multi-morbidity, South Africa, Disability, Chronic disease, Hypertension

## Abstract

**Background:**

Alongside the global population ageing phenomenon, there has been a rise in the number of individuals who suffer from multiple chronic conditions. Taking the case of South Africa, this study aims, first, to investigate the association between multi-morbidity and disability among older adults; and second, to examine whether hypertension (both diagnosed and undiagnosed) mediates this relationship. Lastly, we consider whether the impact of the multi-morbidity on disability varies by socio-demographic characteristics.

**Methods:**

Data were drawn from Wave 1 (2007–08) of the South African Study on Global Ageing and Adult Health. Disability was measured using the 12-item World Health Organisation Disability Assessment Schedule (WHODAS) 2.0. Scores were transformed into a binary variable whereby those over the 90^th^ percentile were classified as having a severe disability. The measure of multi-morbidity was based on a simple count of self-reported diagnosis of selected chronic conditions. Self-reports of diagnosed hypertension, in addition to blood pressure measurements at the time of interview, were used to create a three category hypertension variable: no hypertension (diagnosed or measured), diagnosed hypertension, hypertension not diagnosed but hypertensive measured blood pressure. Interactions between the number of chronic diseases with sex, ethnicity and wealth were tested. Logistic regression was used to analyze the relationships.

**Results:**

25.4% of the final sample had one and 13.2% two or more chronic diseases. Nearly half of the respondents had a hypertensive blood pressure when measured during the interview, but had not been previously diagnosed. A further third self-reported they had been told by a health professional they had hypertension. The logistic regression showed in comparison to those with no chronic conditions, those with one or two or more had significantly higher odds of severe disability. Hypertension was insignificant and did not change the direction or size of the effect of the multi-morbidity measure substantially. The interactions between number of chronic conditions with wealth were significant at the 5% level.

**Conclusions:**

The diagnosis of multiple chronic conditions, can be used to identify those most at risk of severe disability. Limited resources should be prioritized for such individuals in terms of preventative, rehabilitative and palliative care.

## Background

In an ageing global population, the prevalence of non-communicable diseases (NCDs) is increasing worldwide [1], leading to new and different pressures on health systems, especially within low and middle income countries (LMICs). Furthermore, there is a rise in the number of individuals who suffer from multiple conditions at the same time, referred to as multi-morbidity. This is closely related to adverse long-term health outcomes, including mortality [2] and a poorer quality of life [3], while putting further burden on countries’ healthcare systems in terms of complications to treatment strategies alongside increased costs [4].

Disability as a health outcome is being increasingly considered within multi-morbidity research. Generally, the negative effect of multi-morbidity on disability is progressively greater with increases in the number of chronic conditions [3, 5, 6]. However, Bayliss et al.’s [7] longitudinal study of increasing disability in the U.S.A, measured through functional status, found only those with 4 or more chronic conditions experienced different outcomes compared to those with no chronic conditions. Whilst studies frequently control for socio-demographic characteristics, the consideration of whether the association between multi-morbidity and disability varies by group membership remains under-researched. Adjustment to the presence of NCDs is likely to be influenced by various aspects of life, such as personality factors, the environment and the resources that individuals have access to. For example, individuals of higher wealth may have greater access to high quality health care or to other environmental resources that facilitate adjustment to disease, and lessen its impact on functional status. The International Classification of Functioning, Disability and Health (ICF), sees that in addition to disease and the physical environment personal characteristics determine disability [8]. An individuals’ style of coping is a personal determinant of the impact that chronic disease has on their functioning and participation through influencing whether they develop effective strategies to manage tasks [9]. Research exists that suggests socio-demographic characteristics are associated with how individuals cope. For example, Keefe et al.’s [10] study of gender differences in coping among those with osteoarthritic knee pain found that women were more likely to use a problem-focused coping style than men. This coping style may mean that women are more able to identify and seize opportunities that reduce the impact of chronic disease on their functional status. Coping strategies in response to pain and chronic disease have also been found to differ between ethnic groups in the U.S.A (for examples, see Bates and Edwards [11] and Njoku et al. [12]).

No common understanding exists surrounding the type of conditions that should be included in a multi-morbidity measure [9]. Hypertension, or high blood pressure, is frequently included. Whilst a common condition, with an estimated global prevalence of 40% among adults aged 25 years and older [1], the inclusion of hypertension is debatable. It has been identified as an important risk factor for non-communicable diseases such as cardiovascular disease, diabetes and pulmonary complications. Globally, approximately 1 in 2 cases of stroke and ischemic heart disease is attributed to hypertension [13]. Subsequently, hypertension instead could be seen as a risk factor for multi-morbidity. Secondly, whilst screening for hypertension is straightforward, it is an asymptomatic condition. This is problematic where measures of multi-morbidity rely on self-reports of conditions as hypertension may only be identified at the same time as related illnesses are diagnosed.

Taking the case of South Africa, this study aims, first, to investigate the association between multi-morbidity and disability among older adults; and second, to examine whether the existence of hypertension (whether diagnosed or undiagnosed) mediates this relationship. Lastly, it considers whether the impact of the multi-morbidity on disability varies by socio-demographic characteristics. Research into multi-morbidity has been concentrated within higher income countries. Nonetheless, results from the South African Study on Global Ageing and Health (SAGE) reveals a considerable prevalence. Among adults aged 50 years and older, 22.5% reported having been diagnosed with two or more of the chronic conditions that were investigated [14]. There is a need to understand the consequences of multi-morbidity in South Africa, especially given the context of limited resources and competing priorities in healthcare provision.

## Methods

### Study design and sample

This paper draws on data from Wave 1 (2007–08) of the South African SAGE. The survey contains a nationally representative sample of 3842 adults aged 50 years and older. Details of the study, including design and sampling, has been described elsewhere [15].

### Measures

The outcome variable, disability, was measured using the 12-item World Health Organisation Disability Assessment Schedule 2.0 (WHODAS 2.0). Past measures of disability have mostly focused on role functioning or limitations in daily activities. In contrast the WHODAS 2.0 reflects the holistic approach to disability taken by the ICF which views disability as a three level concept consisting of bodily impairments, limitations in activities and restriction in participation [16]. Each item assesses difficulties in different aspects of life using a Likert scale ranging from 0 (no difficulty) to 4 (extreme difficulty) based on a recall period of 30 days preceding the survey. Summary scores were computed through summing the scores assigned to each of the items and converted to 0 to 100 scale. Higher WHODAS scores indicate greater disability. This continuous score was dichotomized to represent the presence of severe disability (0 no, 1 yes) using the 90% percentile score as a cut-off as recommended by Von Korff et al. [17] and used in previous research of disability in older adults [18].

Multi-morbidity was defined as the co-existence of two or more diseases [19]. The occurrence of chronic conditions was self-reported by participants in the SAGE in response to being asked if they have been diagnosed with/or told by a health professional that they have had specific conditions. Conditions considered were cataracts, depression, asthma, chronic lung disease, diabetes, angina, stroke and arthritis. The measure of multi-morbidity was based on simple count. Small group sizes meant those who had two or more chronic conditions were collapsed into a single category. There were 394 individuals (12.9% of the final sample) who had two or more chronic diseases, and of these 122 (4.0% of the final sample) had three or more chronic conditions.

A diagnosis of hypertension was self-reported by participants in response to the question ‘*have you ever been diagnosed with high blood pressure (hypertension)?’.* Additionally, systolic and diastolic blood pressure was measured three times at the time of interviewing using a wrist blood pressure monitor placed on the right arm or wrist of the seated participant [15]. Based on the average of the last two readings, hypertension was defined as a systolic blood pressure ≥ 140 mmHg and/or diastolic blood pressure ≥ 90 mmHg. Using this information, a four-category variable was created: self-reported diagnosis of hypertension and measured blood pressure hypertensive, self-reported diagnosis of hypertension and measured blood pressure normal, no self-reported diagnosis of hypertension and measured blood pressure hypertensive, and no self-reported diagnosis of hypertension and measured blood pressure normal. Both self-reported and measured hypertension was used as there is likely to be a substantial number of individuals who have high blood pressure but who are not aware of this, while those who have been diagnosed may be on drugs to reduce their blood pressure so will not be measured as being hypertensive. The category ‘self-reported diagnosis of hypertension and measured blood pressure normal’ was collapsed with ‘self-reported diagnosis and measured blood pressure hypertensive’ to create a category representing diagnosed hypertension. It was chosen to collapse these categories, as the mean WHODAS score and number of chronic diseases of the two groups did not differ greatly, whilst there were larger differences to other hypertension categories.

Additional covariates tested for an association with disability included sex, age, marital status, wealth, education, ethnicity, region of residence and rural or urban residence. Wealth quintiles as provided by SAGE were used. These estimate wealth using data pertaining to household ownerships of durable groups (for example, a bicycle, internet access in the home and a refrigerator, dwelling characteristics (for example, type of floors and walls) and access to services such as improved water). The SAGE uses Bayesian post-estimation methods to arrange households on an asset ladder [as described by Arokiasamy et al. [20]].

### Statistical analysis

For the purpose of this paper the analysis was restricted to those aged 50 years and older and for whom complete data for all variables of interest were available. This restricted the final sample to 3,055. In accordance to the WHODAS 2.0 manual, if only a single item in the WHODAS 2.0 had a missing response the mean value of the remaining items was assigned to this. The WHODAS 2.0 score was not calculated when there was missing information on more than one item. After this imputation, 2.9% of the individuals (*n =* 112) aged 50 years and older in the original sample had missing WHODAS 2.0 scores. In terms of hypertension 5.4% (*n =* 206) had missing data either in the form of blood pressure measurements at the time of the interview or their self-reported diagnosis status. The exception to this is those who self-reported having been diagnosed with hypertension but had missing data on blood pressure measurements at the time of the interview. These individuals were included in the ‘diagnosed hypertension’ category. 14.5% (*n =* 556) of the original sample of older adults had missing data on ethnicity. Chi-square analysis revealed the non-reporting of ethnicity was significantly associated with sex, but no other social or demographic characteristics. All regression analyses were conducted firstly excluding those with missing values for ethnicity, and secondary including missing as a response category to ethnicity. No substantial differences were found in the results. Missing data for multi-morbidity was 4.6% (*n =* 177), whilst missing data for all other variables equaled less than 2%.

Logistic regression was used to analyze the relationship between multi-morbidity and disability, both univariately (model 1) and after controlling for fixed demographic characteristics (model 2) and social characteristics (model 3). Hypertension was added in model 4 to assess whether it mediates the relationship between disability and multi-morbidity. Lastly, interactions were tested, to assess whether the effect of multi-morbidity differs by the social and demographic characteristics of sex, ethnicity or wealth (only the significant interaction terms were retained in the model and shown in the final output). Odds ratios (ORs) are presented with 95% confidence intervals. All statistical analyses were conducted using STATA software version 14 (Stata Corp. Inc, TX, USA). Survey design effects were controlled for using the svyset command. Sensitivity analysis was conducted running the models using the 80% percentile on the WHODAS 2.0 scores as a cut-off for severe disability. This did not change the conclusions of the results.

## Results

The 90^th^ percentile, used in this study as a cut-off for severe disability, had a WHODAS 2.0 score of 45 and above. This is similar to the ICF’s Disability levels that define severe disability as a score on a standardized instrument on self-reported difficulty for an activity/participation over 50% (score 50 on WHODAS 2.0) and up to 95% of the score range [8]. Using the ICF levels and WHODAS-2.0, Almazán-Isla et al’s. [21] study of disability among those aged 50 years and older in Cinco Villas, Spain, classified 7.7% of the sample as having a severe or complete disability; a figure that is only 2.3 percentage points lower than in our sample.

In this study, participants with severe disability were significantly more likely to be older, of Indian or Asian ethnicity, separated, divorced or widowed, have a low level of education (primary or none), belong to the poorest or poor wealth quintile, live in rural areas and be resident in the Free State, KwaZulu-Natal or Limpopo regions (Table 1). The majority of the sample (61.4%) had none of the chronic diseases asked about in the SAGE, whilst 25.4% had one and 13.2% two or more chronic diseases. Table 1 shows that the percentage of those with severe disability increases with the number of chronic diseases (*χ* ^2^:*p <*0.001). A fifth of those with two or more chronic conditions have severe disability, compared to 14.8% of those with a single chronic disease and 7.2% with no chronic disease. This pattern of results, whereby the percentage with disability becomes progressively greater with increases in the number of chronic conditions, has been observed previously. For example, pooled data from Wave 1 of the SAGE conducted in China, Ghana, India, Mexico, Russia and South Africa found that 7.1% of those with no chronic diseases reported any limitations in their daily activities. This increased to 58.7% for those with four or more chronic diseases [20]. Nearly half (*n =* 1475) of the final sample had a blood pressure that was measured as hypertensive but had not been diagnosed whilst one-third (*n =* 941) self-reported that they had been told by a health professional that they had hypertension. A greater percentage of those who had been diagnosed with hypertension (15.3%) were severely disabled compared to those with measured hypertension but had no diagnosis (9.4%) or no hypertension (7.5%). The high prevalence of hypertension is similar to levels reported by the SAGE for Russia (71.1%), and considerably higher than figures for Mexico (58.2%), India (32.3%), Ghana (57.1%) and China (59.5%) [22]. Similarly to our findings, Lloyd-Sherlock [22] reports that 38% of older South Africans are aware of their positive hypertension status, a figure that is comparable to China (42.7%) and Mexico (44.6%) but lower than Russia (72.1%) and higher than India (27.8%).

**Table 1:** Prevalence of severe disability by background characteristics

Characteristic	n (final sample)	Severe disability (%)
Number of chronic diseases***		
None	1870	7.19
One	791	14.77
Two or more	394	20.30
Age (years)***		
50-59	1342	3.83
60-69	984	13.62
70 plus	729	24.48
*Sex*		
Male	1210	9.51
Female	1845	11.74
Ethnicity*		
Black	1895	11.46
White	242	4.87
Coloured	635	8.79
Indian/Asian	283	20.43
Marital Status**		
Married	1546	8.58
Divorced/widowed/ separated	1084	15.79
Never married	425	8.33
Education**		
None/less than primary	1539	13.39
Primary	731	12.77
Secondary and higher	785	5.12
*Wealth*		
Poorest	573	15.54
Poor	613	13.45
Middle	621	8.50
Rich	622	8.01
Richest	626	8.58
Residence*		
Urban	2049	8.91
Rural	1006	14.49
Region***		
Eastern Cape	731	3.70
Free State	161	11.56
Gauteng	532	5.13
KwaZulu-Natal	451	26.04
Limpopo	245	18.33
Mpumalanga	145	9.32
North-West	310	5.22
Northern Cape	259	8.84
Western Cape	221	6.08
Hypertension**		
No hypertension	639	7.50
Hypertension diagnosed	941	15.30
Hypertension measured but not diagnosed	1475	9.42
Total	3055	10.84

*p value <0.05; **p value < 0.01; ***p value < 0.001

The logistic models, showing the unadjusted and adjusted relationships between the presence of chronic diseases and disability, are shown in Table 2. The number of chronic diseases was significantly associated with disability in the univariate model (model 1) and after controlling for social and demographic characteristics (model 2 and 3). ‘No chronic disease’ was considered the reference category, and in all three models the ORs of being severely disabled increases with the number of chronic conditions. The 95% confidence intervals of the ORs of the categories ‘1 chronic disease’ and ‘2+ chronic diseases’ overlap. Model 4 presents the regression analysis which tests whether the association between disability and number of chronic diseases is mediated by hypertension. Those with diagnosed hypertension or those with a hypertensive blood pressure who have not been diagnosed do not have significantly higher odds of being severely disabled compared with those with no hypertension. In order to assess whether the addition of hypertension into the model changed the size of the coefficients for the number of chronic conditions, the average marginal effects (AMEs) were calculated for models 3 and 4 (output not shown). The addition of hypertension did not substantially change the size of the AMEs of those with at least one chronic condition. For those with one chronic condition the AME was 0.043 in model 3 compared to 0.041 in model 4, while for those with two or more chronic conditions the AMEs were 0.075 and 0.068 for models 3 and 4 respectively.

**Table 2:** Odd Ratios of being severely disabled among older adults in South Africa (unadjusted and adjusted models)

	Model 1		Model 2		Model 3		Model 4		Model 5	
Variable	OR	95% CI	OR	95% CI	OR	95% CI	OR	95% CI	OR	95% CI
*Number of chronic diseases*										
None	1		1		1		1		1	
One	2.236**	1.394–3.586	2.387***	1.468–3.884	2.413***	1.509–3.861	2.330**	1.422–3.820	4.254**	1.668–10.848
Two or more	3.288***	1.866–5.794	3.417***	1.891–6.176	3.559***	1.919–6.600	3.272***	1.681–6.373	6.641***	2.226–19.814
*Age (years)*										
50–59			1		1		1		1	
60–69			3.797***	2.225–6.481	4.238***	2.336–7.689	4.187***	2.308–7.596	4.412***	2.417–8.052
70 plus			8.935***	5.193–15.375	11.963***	6.250–22.901	11.669***	6.111–22.284	12.592***	6.538–24.251
*Sex*										
Male			1		1		1		1	
Female			1.043	0.653–1.664	0.887	0.511–1.540	0.862	0.508–1.462	0.855	0.501–1.459
*Ethnicity*										
Black			1		1		1		1	
White			0.241**	0.096–0.601	0.396	0.122–1.281	0.400	0.120–1.339	0.437	0.135–1.413
Coloured			0.504*	0.262–0.969	1.454	0.778–2.720	1.452	0.774–2.726	1.513	0.804–2.848
Indian/Asian			1.376	0.599–3.165	0.898	0.334–2.410	0.906	0.338–2.425	0.966	0.354–2.634
*Marital status*										
Never married					1		1		1	
Married/cohabiting					1.050	0.489–2.254	1.059	0.497–2.259	0.942	0.451–1.965
Separated/widowed/divorced					1.241	0.635–2.424	1.250	0.642–2.436	1.110	0.571–2.159
*Education*										
None or less than primary					1		1		1	
Primary					1.183	0.719–1.947	1.197	0.730–1.962	1.159	0.707–1.899
Secondary or higher					0.528	0.249–1.118	0.524	0.248–1.105	0.536	0.260–1.107
*Wealth*										
Poorest					1		1		1	
Poor					0.827	0.435–1.570	0.829	0.440–1.156	1.676	0.729–3.854
Middle					0.357**	0.194–0.658	0.352**	0.191–0.646	0.395	0.153–1.024
Rich					0.331**	0.172–0.635	0.329**	0.172–0.630	0.357**	0.141–0.905
Richest					0.428	0.181–1.012	0.424	0.180–1.000	0.632	0.187–2.133
*Residence*										
Urban					1		1		1	
Rural					0.971	0.589–1.602	0.975	0.589–1.614	0.943	0.575–1.544
*Region*										
Eastern Cape					1		1		1	
Free State					5.785***	2.492–13.433	5.708***	2.475–13.161	5.517***	2.372–12.835
Gauteng					2.562*	1.153–5.695	2.418*	1.091–5.358	2.384*	1.077–5.275
KwaZulu-Natal					17.398***	8.546–35.419	16.901***	8.358–34.176	16.768***	8.265–34.019
Limpopo					9.612***	4.369–21.146	9.468***	4.305–20.824	9.182***	4.142–20.358
Mpumalanga					5.635***	2.198–14.446	5.728***	2.247–14.602	5.546**	2.048–15.019
North-West					1.887	0.902–3.948	1.858	0.884–3.902	1.891	0.905–3.955
Northern Cape					4.192***	1.961–8.961	4.107***	1.919–8.792	3.953**	1.811–8.507
Western Cape					1.785	0.709–4.495	1.753	0.697–4.414	1.743	0.708–4.296
*Hypertension*										
No							1		1	
Hypertension diagnosed							1.365	0.749–2.489	1.378	0.753–2.523
Measured but not diagnosed							1.157	0.684–1.956	1.179	0.692–2.007
Wealth*no of chronic conditions										
Poor*one chronic disease									0.150**	0.040–0.559
Poor* two plus chronic diseases									0.311	0070–1.382
Middle*one chronic disease									1.193	0.326–4.366
Middle*two plus chronic diseases									0.287	0.0522–1.573
Rich*one chronic disease									0.802	0.199–3.236
Rich*two plus chronic diseases									0.578	0.107–3.200
Richest*one chronic disease									0.357	0.077–1.648
Richest*two chronic diseases									0.476	0.076–2.960

*p value <0.05; **p value < 0.01; ***p value < 0.001

**Fig. 1:**
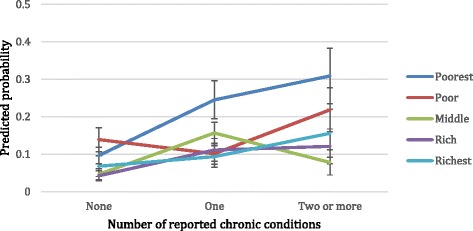
Predicted probability of being severely disabled by wealth status and number of chronic diseases. Note: probabilities refer to when holding all other variables in the model constant at the reference category

In the final stage, interactions between the number of chronic conditions with sex, ethnicity and wealth were introduced into the model individually. Only the interaction between wealth and number of chronic diseases was significant and retained in the model (model 5 in Table 2). The predictive probabilities were plotted to ease the interpretation of the interaction (Fig. 1). Figure 1 reveals that when focusing on the poorest wealth quintile, its association with severe disability differs according to the number of chronic diseases reported. With each increase in the number of chronic diseases reported, there is an increase in the predicted probability of being severely disabled amongst those in the poorest wealth quintile. This pattern does not hold true for the other four wealth quintiles.

In the final model (model 5), age and region was also found to be significantly associated with severe disability. The association found with age was as expected. In comparison to those living in the Eastern Cape, those living in Free State, Guateng, KwaZulu-Natal, Limpopo, Mpumalanga and Northern Cape have higher odds of being severely disabled. In some cases, the odds are dramatically raised, with those in KwaZulu-Natal almost 17 times more likely to have a severe disability than those in the Eastern Cape. Although the confidence intervals are wider due to smaller group sizes, this is a clear and highly significant result.

## Discussion

In this study the association between multi-morbidity and disability among older adults in South Africa was examined. Furthermore, this study provided new information on the effect that hypertension and socio-demographic characteristics have on this relationship within the country.

The results show that the prevalence of severe disability among older adults in South Africa differs according to the number of chronic conditions diagnosed, with this being greatest among those with multi-morbidity. Disability among older adults is a financial challenge for health systems [23, 24]. In South Africa the additional challenge of a quadruple health burden, in terms of injuries, non-communicable disease and HIV-AIDS alongside other communicable disease [25], further poses the question of how to best allocate constrained health resources. Our results suggest that the diagnosis of chronic conditions, especially two or more, can be used to identify those most at risk of severe disability, and that the limited resources available should be prioritized for such individuals in terms of preventative, rehabilitative and palliative care.

In contrast to the majority of previous research, in this study hypertension was not classified as a chronic condition that was considered as part of the multi-morbidity measure. Instead, this study considered whether hypertension, categorized according to diagnosis and measured blood pressure, could explain the relationship between multi-morbidity and disability. The results showed that hypertension was not a mediator. It was also found that an interaction between number of chronic diseases and hypertension status was not significant, suggesting that the association between disability and multi-morbidity does not vary by an individual’s hypertension status. Furthermore, sensitivity analysis revealed that including hypertension in the count of chronic conditions did not substantially change the association with disability. When including those with hypertension in the count of multi-morbidity, 23% (rather than 11%) of older adults reported two or more chronic conditions, from the selected diseases. To help explain these results further descriptive analysis was conducted looking at the association between hypertension status and the diagnosis of other chronic conditions (Table 3). A higher percentage of those who had been diagnosed with hypertension, regardless of their measured blood pressure, had been diagnosed with other chronic conditions. This link between hypertension and other chronic conditions suggests that hypertension is only diagnosed when individuals seek health care due to other chronic conditions. This finding questions the inclusion of hypertension in measures of multi-morbidity in other research.

**Table 3:** The percentage diagnosed with selected chronic diseases by hypertension status

	%							
	Arthritis	Stroke	Angina	Diabetes	Lung disease	Asthma	Depression	Cataracts
No hypertension	18.30	1.13	5.85	4.55	3.33	3.16	3.76	2.78
Hypertension diagnosed and measured	35.61	8.91	6.25	21.44	4.02	7.35	3.63	8.92
Hypertension measured but not diagnosed	18.83	2.24	3.43	4.32	1.54	3.05	1.40	1.18
Normal blood pressure but diagnosed with hypertension	38.90	3.91	12.32	15.65	7.72	14.43	7.55	12.56

The World Health Organisation identifies hypertension as a major contributor to disability-adjusted life years globally [1]. The insignificance of hypertension in our logistic models suggests that it influences disability through other chronic diseases for which it is a risk factor. High blood pressure was very common among the participants in this study, even though the majority had not been formally diagnosed with hypertension. The rate of treatment and control of blood pressure in South Africa, as well as other LMICs, is even lower [26]. Early diagnosis and the effective treatment of hypertension are core strategies for disability prevention. Nonetheless, the asymptomatic nature of high blood pressure, combined with the affordability and availability of health care in South Africa, are barriers to this [27]. Possible interventions in this area might include the use of community health workers to increase diagnosis and continued treatment for those whom access to health care is problematic, as well as a targeted national campaign for older adults which highlight the causes and risks of hypertension.

The analysis of the interactions between the number of chronic conditions and wealth, gender and ethnicity, found only wealth to be significant. The probability of those in the poorest wealth quintile being severely disabled increased with the number of chronic conditions reported, a pattern not found when the number of chronic conditions was interacted with the other wealth quintiles. It could be that the poorest are unable to access resources that allow them to adjust to disease, and lessen its impact on functional status. Our findings concerning gender differ to that found by Garin et al. [5] among older adults in Spain which suggest a female disadvantage in the impact of chronic diseases when one or two conditions are present, but the disappearance of this difference once a certain level of multi-morbidity is reached. The limited number of studies in different environments that consider whether the impact of multi-morbidity differs according social factors calls for further research in this area.

In this study only the association between the number of chronic conditions and disability was considered. It should be noted, however, the effect of multi-morbidity may not be simply additive, but specific combinations of diseases have greater associations with disability than others and the effect of two diseases may not equal the sum of the effect of each one individually [5, 6, 20, 28, 29]. For example, the specific combination of diseases may be important and the interaction of two diseases may result in an association with disability which is greater or less than the sum of the effect of the diseases individually. This was found in McDaid et al’s. [6] study of multiple chronic diseases in Northern Ireland and the Republic of Ireland. The interactions between cardio-vascular disease and diabetes, cardio-vascular disease and chronic pain and lung disease and chronic pain revealed that the effect of the second disease on disability was significantly less in the presence of the first disease than would be the case in the absence of the first disease [6].

This study had several other limitations that merit recognition. As acknowledged by Phaswana-Mafuya et al. [14], reliance on self-reports of disease could result in underestimation of the prevalence of multi-morbidity. Individuals may have conditions which they have not been officially diagnosed with, or remain unaware of the symptoms and their significance. Furthermore, the SAGE only enquires about the diagnosis of a limited set of chronic conditions, and the exclusion of conditions such as cancer from the questionnaire is likely to have impacted on the level of multi-morbidity of chronic diseases found in this population. The inaccuracies with measuring hypertension in field-research have been noted previously [26]. Basing hypertension status on three measurements taken within a single hour is problematic where individuals may have just exercised or are nervous. In addition to accuracy of reporting, our measurement of multi-morbidity also has shortcomings in that it fails to take account of the severity of conditions. This study was unable to consider this due to data limitations.

## Conclusion

There are high levels of multi-morbidity in the older population in South Africa, with almost 13% of those aged over 50 reporting more than one diagnosed disease, selected from a relatively narrow list of potential NCDs. This is closely related to the high level of disability amongst the older population, with 11% of the respondents having a WHODAS score of 45 or over. This level is close to the cut off for ‘severe disability’ as defined by the International Classification of Functioning and Disability [8]. Furthermore, levels of hypertension in South Africa are substantial, especially when undiagnosed cases are added to those which have already been diagnosed. Whilst in South Africa several studies have considered the prevalence and socio-demographic associations with multi-morbidity [14, 30, 31], research on the consequences remained under-researched.

The link between multi-morbidity and disability is clear and understandable. The relationship is not affected by the addition of socioeconomic factors, indicating that the relationship is strong and robust. Multi-morbidity was defined in the absence of hypertension to reflect the fact that high blood pressure is on the causal pathway for many NCDs. Many individuals are likely to receive the diagnosis for hypertension and the related NCD at the same time, and hence treating these as separate diseases (and therefore multi-morbidity) is problematic. This research has therefore contributed to the understanding of multi-morbidity, disability and hypertension in South Africa.

Understanding the links between socioeconomic status, the diagnosis of illness and disability will aid the South African health system in order to provide adequate care for its older population. Clearly those with more than one NCD must be targeted for health interventions, in terms of preventative, rehabilitative and palliative care in order to ensure that disability is kept to a minimum and managed. To further aid the distribution of scarce resources in the country additional research is required on exploring the order at which individuals are diagnosed as having an NCD and how disability temporally links to these diagnoses.

## Abbreviations

AME: Average marginal effects
ICF: International classification of functioning, disability and health
LMICs: Low and middle income countries
NCDs: Non-communicable disease
OR: Odds ratio
SAGE: Study on global ageing and adult health
WHO: World health organisation
WHODAS 2.0: World health organisation disability assessment schedule version 2
